# Your mitochondria are what you eat: a high‐fat or a high‐sucrose diet eliminates metabolic flexibility in isolated mitochondria from rat skeletal muscle

**DOI:** 10.14814/phy2.13207

**Published:** 2017-03-22

**Authors:** Wenche Jørgensen, Kasper A. Rud, Ole H. Mortensen, Lis Frandsen, Niels Grunnet, Bjørn Quistorff

**Affiliations:** ^1^ Department of Biomedical SciencesSection of Cellular and Metabolic ResearchFaculty of Health and Medical SciencesUniversity of CopenhagenCopenhagenDenmark

**Keywords:** High fat feeding, high sucrose feeding, metabolic flexibility, PDH‐flux, PDH‐P, pyruvate dehydrogenase, *Randle* glucose‐fatty‐acid‐cycle, skeletal muscle mitochondria, substrate choice, TCA‐flux

## Abstract

Extreme diets consisting of either high fat (HF) or high sucrose (HS) may lead to insulin resistance in skeletal muscle, often associated with mitochondrial dysfunction. However, it is not known if these diets alter normal interactions of pyruvate and fatty acid oxidation at the level of the mitochondria. Here, we report that rat muscle mitochondria does show the normal *Randle‐type* fat‐carbohydrate interaction seen in vivo. The mechanism behind this metabolic flexibility at the level of the isolated mitochondria is a regulation of the flux‐ratio: pyruvate dehydrogenase (PDH)/*β*‐oxidation to suit the actual substrate availability, with the PDH flux as the major point of regulation. We further report that this regulatory mechanism of carbohydrate‐fat metabolic interaction surprisingly is lost in mitochondria obtained from animals exposed for 12 weeks to a HF‐ or a HS diet as compared to rats given a normal chow diet. The mechanism seems to be a loss of the PDH flux decrease seen in controls, when fatty acid is supplied as substrate in addition to pyruvate, and *vice versa* for the supply of pyruvate as substrate to mitochondria oxidizing fatty acid. Finally, we report that the calculated TCA flux in the isolated mitochondria under these circumstances shows a significant reduction (~50%) after the HF diet and an even larger reduction (~75%) after the HS diet, compared with the chow group. Thus, it appears that obesogenic diets as those applied here have major influence on key metabolic performance of skeletal muscle mitochondria.

## Introduction

Insulin resistance may be induced in liver and skeletal muscle in various experimental animal models by high‐fat (HF) or high‐sucrose (HS) diets (Kim et al. 1999; Buettner et al. 2007) and many studies indicate that the mitochondrial function is changed when insulin resistance is present (Kelley et al. 2002; Petersen et al. 2004; Asmann et al. 2006; Mogensen et al. 2007; Jorgensen et al. 2012).

Both diets mentioned may be considered extreme and results in a significant change in the normal balance between substrates presented to cell intermediary metabolism. Typically it also involves a substrate overload concerning the substrate moiety in excess in the particular diet, that is, fatty acids or sucrose (glucose and fructose). Thus, it is likely that this type of changed metabolic conditions could affect the normal regulatory mechanisms of the cells, in particular in tissues like liver and muscle, with high‐energy turnover. In any event, such extreme diet can be expected to put the metabolic flexibility of the tissues to the test. Yet, little information on intracellular substrate interaction is available, in particular under conditions of substrate overload (Koves et al. 2008), where the mitochondria must make a choice between acetyl CoA delivered by either the pyruvate dehydrogenase (PDH) or the *β*‐oxidation. The mechanism of this substrate choice at the cellular level is poorly understood and is only rarely studied in isolated mitochondria (Kerbey et al. 1976; Hue and Taegtmeyer 2009). However, the foundation was laid ~50 years ago by Randle et al. (Randle et al. 1963; Randle 1998) coining the concept of the *“glucose‐fatty acid cycle”* which states that increased plasma fatty acid promotes *β*‐oxidation and suppresses glucose oxidation. The reverse phenomenon, inhibition of *β*‐oxidation by excess carbohydrate, was later demonstrated by McGarry et al. (1977). The *glucose‐fatty acid cycle,* the so called *Randle‐effect,* as observed at the whole body level is sometimes loosely also referred to as “metabolic flexibility” (Kelley et al. 1999; Galgani et al. 2008) and lack of metabolic flexibility has been associated with type 2 diabetes (Kelley et al. 1999, 2002; Kelley and Mandarino 2000; Bergman et al. 2007; Galgani et al. 2008; Noland et al. 2009). Accordingly, the substrate choice event in the cell can be considered an important and most probably dynamic adaptation of the mitochondrial metabolic pathways, in particular the interactions between the pyruvate dehydrogenase, the *β*‐oxidation and the TCA cycle flux, in response to shifting substrate availability. Importantly, it may be concluded that if such pathway regulatory interactions can be demonstrated at the level of isolated mitochondria, it must be operating purely by inter‐pathway regulations without the effects of hormonal signaling or mitochondrial‐cytosolic interactions.

In the present paper we have therefore addressed the following questions:

1. Does isolated mitochondria from skeletal muscle of normal rats display a carbohydrate–fat substrate interaction as seen in vivo in the intact animal (a Randle‐type phenomenon)?
2. Is this substrate interaction ‐ if present ‐ affected by prior exposure of the animal to a high‐fat or a high‐sucrose diet, and how is it regulated?

## Materials and Methods

### Reagents

Unless otherwise stated, reagents were purchased from Sigma‐Aldrich, Inc., and were of analytical grade or better.

### Animals

Pregnant Wistar Hannover GALAS rats (bodyweight approximately 200 g) were purchased from Taconic (Ejby, Denmark). The male pups from these rats were randomly assigned to the following three diets for 12 weeks after weaning: (1) a high‐sucrose diet (HS), (2) a high‐fat diet (HF) and (3) normal rat chow. The HS diet consisted of 70E% carbohydrate (34.5% sucrose, 32% corn starch, 3.5% maltodextrin 10), 20E% protein and fat; cat. no: D12450B. The HF diet consisted of 60E% fat, 20E% carbohydrate (6.8E% sucrose, 0.9%, 12.3E% maltodextrin), 20E% protein; cat. no: D12492. Both diets were purchased from *Open source Diets* (New Brunswick). The chow diet was Altromin 1310 (standard breading diet at Panum Animal Facility, University of Copenhagen, Denmark).

Animals of all groups were sacrificed and skeletal muscle tissue sampled 12 weeks after weaning.

### Preparation of isolated muscle mitochondria

Mitochondria was prepared as described in (Fritzen et al. 2007). In brief: Quadriceps muscle was dissected from both hind legs while the rat was under general anesthesia (Hypnorm/Dormicum, 0.3 mL/100 g). The muscle tissue was kept in cold KCl‐buffer (100 mmol/L KCl, 50 mmol/L Tris‐Base, 5 mmol/L MgSO_4_·7H_2_O, 1 mmol/L EDTA, pH 7.4 at 4°C). This buffer was decanted from the biopsy and replaced with 20 mL ice cold buffer ATP‐buffer: KCl buffer with 1 mmol/L ATP and 0.5% BSA with 20 mg proteinase (Subtilisin A, 7–15 units/mg). After 10 min the tissue was washed three times in the same buffer followed by homogenization (glass‐teflon Potter Elvehjem). The homogenate was centrifuged at 380***g*** for 5 min at 4°C in order to remove connective tissue and the supernatant further centrifuged at 5340***g*** for 10 min at 4°C. The pellet was resuspended in 8 mL KCl‐buffer, and this suspension centrifuged at 6700***g*** for 10 min at 4°C. The final mitochondrial pellet was resuspended in 1 mL of MSTP_i_‐medium: 225 mmol/L mannitol, 75 mmol/L sucrose, 20 mmol/L Tris‐Base, 10 KH_2_PO_4_, 0.5 mmol/L EDTA, pH 7.0. This suspension, containing 3–5 mg protein per mL, was used for measurements of respiration in the oxygraphs as well as for substrate oxidation rates in parallel incubations. Mitochondrial preparations were tested for outer membrane integrity by observing the changes of the addition of cytochrome c during state 3 respiration. Only preparations with negligible changes were included in the study.

#### Mitochondrial respiration

Mitochondrial function was tested as described in (Jorgensen et al. 2012). In brief: Oroboros Oxygraph‐2K instruments (Oroboros Inc., Austria) were used, operating eight oxygraph chambers in parallel at 25°C. 10 *μ*L mitochondrial suspension was added to 2 mL of medium in each chamber. Stirrer speed was 600 rpm. Approximately 5 min after the mitochondria were introduced into the chambers, malate and pyruvate or palmitoyl carnitine (PC) was added to concentrations of 2 mmol/L, 0.5 mmol/L and 10 *μ*mol/L, respectively. Once a steady state 4 respiration was observed, ADP (3 mmol/L) was added to obtain a state 3 respiration. The final addition was succinate (5 mmol/L).

The respiratory control ratio (RCR) was calculated as the state 3/state 4 ratio. The VO_2max_ was taken as the state‐3 respiration with the addition of succinate, in order to include also the complex II contribution to oxygen consumption.

#### Pyruvate and palmitoyl carnitine (PC) oxidation

In experiments parallel with the oxygraph measurements pyruvate as well as PC oxidation was measured as the rate of ^14^CO_2_ formation from [1‐^14^C]pyruvate, and for palmitoyl carnitine as the sum of formation of ^14^CO_2_ and ^14^C‐labeled acid soluble metabolites (^14^C‐ASM) from [1‐^14^C] palmitoyl carnitine. Radioisotopes were obtained from Perkin Elmer, Boston, MA.

Mitochondria (approximately 0.5 mg of mitochondrial protein) were incubated at 25°C in a conical flask (with a center well) in 1 mL MSTP_i_ buffer with 2 mmol/L malate and 3 mmol/L ADP. At zero time [1‐^14^C]pyruvate or [1‐^14^C]palmitoyl carnitine was added to final concentrations of 0.5 mmol/L/0.06 *μ*Ci and 0.01 mmol/L/0.05 *μ*Ci, respectively. In experiments with both substrates present, unlabeled pyruvate or palmitoyl carnitine was added to 0.5 mmol/L and 0.01 mmol/L, respectively. The reaction was stopped after 10 min by injecting perchloric acid (PCA) in the main chamber well (0.7 mol/L PCA final concentration) which releases the CO_2_ produced. The center well contained 200 *μ*L 0.5 mol/L KOH on a filter paper absorbing the CO_2_ quantitatively during a furthermore 2 h incubation at 25°C of the still sealed incubation flasks. Subsequently the filter paper was removed from the flasks and eluted in water overnight and the radioactivity measured in Ultima Gold scintillation liquid (Perkin Elmer, Boston, MA). ^14^C‐ASM was determined on the supernatant of the acidified incubation mixture of the main chamber after centrifugation for 5 min at 20,000 g. As blanks were used zero‐time incubations.

### Oral glucose tolerance test/Insulin tolerance test

#### OGTT

At age 9 weeks ‐ following an overnight fast (16–18 h), rats were weighed, a tail vein punctured using a lancet (BD Microtainer, Contact‐Activated Lancet, 2.0 mm × 1.5 mm, BD biosciences, Erembodegem, Belgium), baseline plasma glucose was measured using plasma calibrated strips (Accu‐Chek Compact Plus, Roche, Basel, Schweiz) and a blood sample (~200 *μ*L) collected for EDTA‐plasma (Microvette CB 300, Sarstedt, Nümbrect, Germany). Rats were gavaged a 2 g/kg body weight dose of glucose from a 22.5% (w/v) glucose solution. At 30, 60, 90, and 120 min after gavages, plasma glucose was measured from fresh tail vein blood samples.

#### ITT

At age 10 weeks ‐ following an overnight fast (16–18 h), rats were weighed and baseline plasma glucose determined on tail vein punctures. Rats were then injected intraperitoneally with 1.2 U/kg insulin (Actrapid, NovoNordisk Inc., Bagsværd, Denmark) from a 0.5 U/mL insulin solution in 0.2% BSA, 0.1 mol/L HCl and plasma glucose measured at 15, 30, 45, 60, and 90 min from fresh tail blood samples as above.

### Western blotting

Isolated rat quadriceps mitochondria (200 *μ*L, 5 mg protein/mL) was centrifuged (20,000***g***) for 10 min at 4°C and resuspended in lysis buffer pH 7.4 (50 mmol/L Tris‐HCl, 150 mmol/L NaCl; 1 mmol/L EGTA; 1 mmol/L EDTA; 0.25% NaDeoxycholate; 1% Triton X‐100) with phosphatase and protease inhibitors: 1 *μ*g/mL Pepstatin A, 1 mmol/L Na_3_VO_4_, 1 mmol/L NaF, Sigma PI #1, Sigma PI #2, Complete Mini (Roche). Mitochondria were frozen in liquid nitrogen, thawed and centrifuged 20,000***g*** for 10 min at 4°C. Volumes corresponding to 13 *μ*g protein were loaded and subjected to SDS‐PAGE in 15 wells NuPage 10% Bis‐Tris precast gels at 125 V for 90 min. Proteins were electroblotted onto PVDF membranes at 30 V for 90 min, then washed in 0.1% Tween 20 in 1xTBS (TBS‐T) and blocked for 1 h in 5% dry skim milk in TBS‐T. Subsequently, membranes were probed with one of following antibodies: Rabbit anti‐PDH‐pSer^293^ (AP 1062) (EMD, Merck, Darmstadt, Germany), mouse anti‐PDH‐E1*α* (MSP03)(MitoScience, Eugene, OR), rabbit anti‐PDK4 (ab89295)(abcam) diluted to 0.25 *μ*g/*μ*L; 0.1 ng/*μ*L; and 0.05 ng/*μ*L, respectively, in 5% Bovine serum albumin over night at 4°C. The next day immunoblots were washed in TBS‐T and incubated for 1 h at room temperature with HRP‐conjugated anti‐rabbit (Dako, P0448) diluted 1:10,000 for PDH‐pSer^293^, and 1:25,000 for PDH‐E1*α* and PDK4 in 5% dry skim milk. After washing, specific binding was detected using Femto (Pierce Technology, Omaha, NE) visualization systems. Quantification was performed using the Image J gel analysis software (open source http://rsbweb.nih.gov). Variations in intensities between gels were corrected according to an inter‐gel standard, prepared from a pool of all experimental samples. All buffers and gels were purchased from Invitrogen (Carlsbad, CA).

### Protein concentration and citrate synthase activity

Protein concentration and citrate synthase (CS) activity in the muscle biopsies and the isolated mitochondria suspensions were determined in 10% homogenates prepared in buffer (50 mmol/L Tris‐HCl; 0.6 mmol/L MnCl_2_; 2 mmol/L citrate; 0.1% Triton x‐100. pH = 7.4) and applying an Ultra Turax homogenizer. Protein concentrations were determined using Lowry's method (Lowry et al. 1951). The CS (EC 4.1.3.7) activity was assayed at 25°C according to (Srere 2012).

### Statistical analysis

Two‐way analysis of variance (ANOVA) was performed on the HF and HS groups, and one‐way ANOVA was used to compare the two groups to the chow group. Both types of ANOVA analyses were followed by a post hoc Tukey HSD test. The statistical analyses were performed using the R program (R 2.9.0, www.r-project.orgClick here to enter text.).The statistical analysis within groups was performed by paired *t*‐tests or by repeated measures ANOVA when appropriate, as further explained in the legends of the tables. The level of significance was set at *P* < 0.05.

## Results

The present experiment compares the effects of a high‐sucrose (HS) and a high‐fat (HF) diet for 12 weeks after weaning against a control group where mothers and pups were fed an ordinary chow diet throughout. Pyruvate and palmitoyl‐carnitine (PC) was used as substrates for the isolated mitochondria, reflecting the mitochondrial component of carbohydrate and fat oxidation, respectively. Thus, PC rather than free fatty acid was used as fat substrate in order to focus on the internal mitochondrial oxidation pathways only, that is, pyruvate dehydrogenase and the *β*‐oxidation pathways, assuming PC transport is not a regulatory factor of the *β*‐oxidation.

### Glucose and insulin tolerance

**Figure 1:**
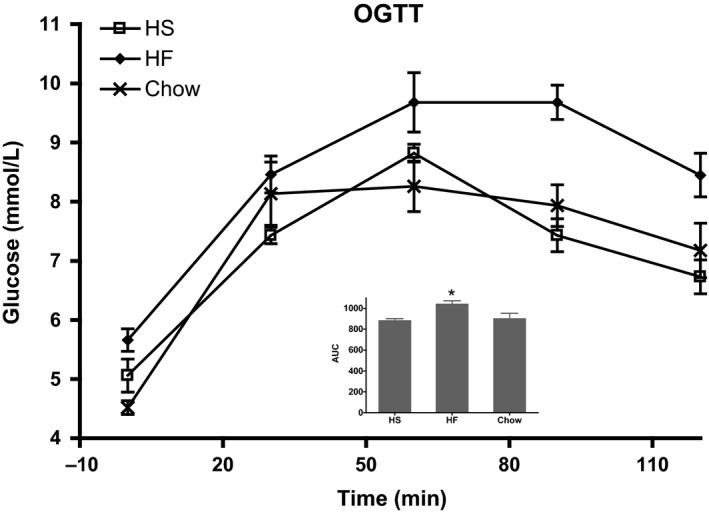
Oral glucose tolerance test. Oral glucose tolerance test is depicted as plasma glucose concentration and as area under the curve (inserted bar graph). Groups are defined by progeny diet. Calculations were performed with 0 mmol plasma glucose per liter as baseline and values in the bar graph are mean ± SD,* n* = 5–6. *Denotes significance with respect to chow, *P* < 0.05.

Results from oral glucose tolerance test (OGTT) of the three groups were expressed as AUC (mean ± SEM; *n* = 5–6): 905 ± 48, 890 ± 14 and 1046 ± 27 (*n* = 5) for Chow, HS and HF groups, respectively. The area under the curve (AUC) increased (*P* < 0.05) for the high‐fat group, while the HS group did not show a significant change compared to the chow group, (Fig. 1). An insulin tolerance test (ITT) did not report any difference between groups (data not shown).

### Oxygen consumption with pyruvate and/or palmitoyl carnitine (PC), with/without succinate

In mitochondria isolated from the quadriceps muscles, the state 4 and state 3 respiration was recorded with either a carbohydrate substrate (pyruvate), a fatty acid substrate (palmitoyl carnitine, PC) or a combination of these two substrates. The complex II contribution to oxygen consumption was further evaluated by adding succinate during state 3 respiration. The latter condition is referred to as the physiological mitochondrial VO_2max_ of the isolated mitochondria. Malate, 2 mmol/L, was present in all oxygraph incubations in order to ensure sufficient level of TCA intermediates.

Oxygen consumption data are presented in Table 1.

**Table 1:** Oxygen consumption in isolated mitochondria of rat quadriceps muscle

Progeny diet	Chow	HS	HF
State 4			
Pyr	9.3 ± 0.5	2.7 ± 0.6^a^^,^ b	5.1 ± 1.0^a^
PC	10.7 ± 1.0	5.3 ± 2.9^a^	7.5 ± 1.3^a^
Pyr + PC	12.6 ± 1.1	5.3 ± 1.8^a^	8.7 ± 3.7^a^
State 3			
Pyr	200 ± 24	77.2 ± 25^a^^,^ c	129 ± 36^a^
PC	97.6 ± 7.5	63.2 ± 24^a^	85.3 ± 26
Pyr + PC	224 ± 19	102 ± 26^a^	156 ± 56^a^
VO_2 max_			
Pyr + succ	256 ± 32^d^	116 ± 26^a^^,^ d	173 ± 47^a^^,^ d
PC + succ	143 ± 11^d^	71.6 ± 24^a^	114 ± 37^d^
Pyr + PC + succ	277 ± 24^d^	134 ± 33^a^^,^ d	200 ± 68^a^^,^ d
RCR			
Pyr	21.6 ± 2.6	32.9 ± 9.0^a^	27.0 ± 9.7
PC	9.19 ± 1.5	9.11 ± 6.6	11.5 ± 3.3
Pyr + PC	18.0 ± 2.9	18.7 ± 9.3	18.3 ± 2.1

Groups are defined by the progeny diet, that is, standard chow (Chow), high sucrose (HS) and high fat (HF) for 12 weeks after weaning. Values are given as mean ± SD (*n* = 5–8) (nmol/[min × mg mitochondrial protein]).

a Significantly different from Chow, *P* < 0.05.

b Significant difference between HF and HS, *P* < 0.05 and

c *P* < 0.1.

d Significantly different from the corresponding state 3 oxygen uptake, *P* < 0.01.

With pyruvate as substrate, the state 4, state 3 and VO_2max_ respiration was lower (*P* < 0.05) in the HF and HS groups, compared to the chow group. With PC as substrate the same pattern was observed in state 4. Yet, during state 3 and VO_2max_ respiration with PC as substrate, the HS group displayed a significantly ~2‐fold decreased (*P* < 0.05) respiration compared with the chow group.

Adding both the carbohydrate and fat substrate (pyruvate and PC), the state 3 respiration was unchanged compared with pyruvate alone in the chow group. However, the state 3 oxygen consumption of the HS and HF groups were significantly lower than the chow group.

The complex II contribution (succinate addition) to the rate of state 3 oxygen consumption was similar in chow and HF animals (30–58 nmol/min/mg mitochondrial protein), but was abolished (no significant oxygen consumption increase by succinate addition) for the HS animals when oxidizing PC. In other words, under conditions of fat oxidation in the mitochondria of the HS group, the control strength of the SDH reaction in the regulation of oxygen consumption would appear to be low. The calculated RCR value during pyruvate oxidation was higher (*P* < 0.05) for the HS group compared to chow, and significantly lower (*P* < 0.05) with PC than with pyruvate as substrate for all groups (Table 1).

### Mitochondrial substrate oxidation rate [pyruvate and palmitoyl carnitine (PC)]

The oxidation rate of [1‐^14^C]pyruvate and [1‐^14^C]palmitoyl carnitine was measured in parallel experiments under state 3 conditions in the same mitochondrial preparations used for the oxygen consumption measurements. Data are reported in Table 2. With pyruvate as substrate, the PDH flux was 93.4 ± 15 nmol (min·mg mitochondrial protein)^−1^ in the chow group, while for the HS and HF groups, the PDH flux rate was 20–50% lower (*P* < 0.05). Adding PC in addition to pyruvate as substrate to the mitochondria caused a 27% reduced pyruvate oxidation rate (*P* < 0.05) in the chow group. Yet, there was no such substrate interaction of PC addition in the HS and HF groups. In other words, the PDH flux was affected by the PC addition only in mitochondria from the control, chow fed animals, while prior exposure of the animal to the high‐fat or the high‐sucrose diets abolished normal *Randle‐type* metabolic flexibility of carbohydrate and fat oxidation interaction. In the reverse experiment (adding pyruvate to mitochondria already oxidizing PC) similar results were observed, that is, a ~20% decreased PC oxidation rate by addition of pyruvate in the chow group and a loss of this substrate interaction in the HS group. Yet, for the HF group, a significant (*P* < 0.05) substrate interaction was in fact present.

**Table 2:** Oxidation rates of [1‐^14^C]‐pyruvate and [1‐^14^C]‐palmitoyl carnitine in isolated mitochondria from rat quadriceps muscle

Progeny diet	Chow	HS	HF
Substrate oxidation			
Pyruvate oxidation	93.4 ± 15	48.2 ± 10^a^	61..2 ± 9.7^a^
Pyruvate oxidation in the presence of PC	68.2 ± 17^b^	44.6 ± 8.3^a^	60.6 ± 5.3^c^
PC oxidation	3.53 ± 1.1	2.84 ± 1.3	3.26 ± 0.78
PC oxidation in the presence of pyruvate	2.89 ± 0.80^d^	2.71 ± 1.2	2.73 ± 0.97^d^

Groups are defined by the progeny diet, that is, standard chow (Chow), high sucrose (HS) and high fat (HF) after weaning. Values are mean ± SD (*n* = 5–8) (nmol substrate/mg protein/min).

a Significantly different from Chow.

b Significant difference between *pyruvate* and *pyruvate* *+ PC*.

c Significantly different from HS.

d Significant difference between *PC* and *PC + pyruvate*.

### PDH, PDH‐P, and PDK; protein amounts versus PDH‐flux

**Figure 2:**
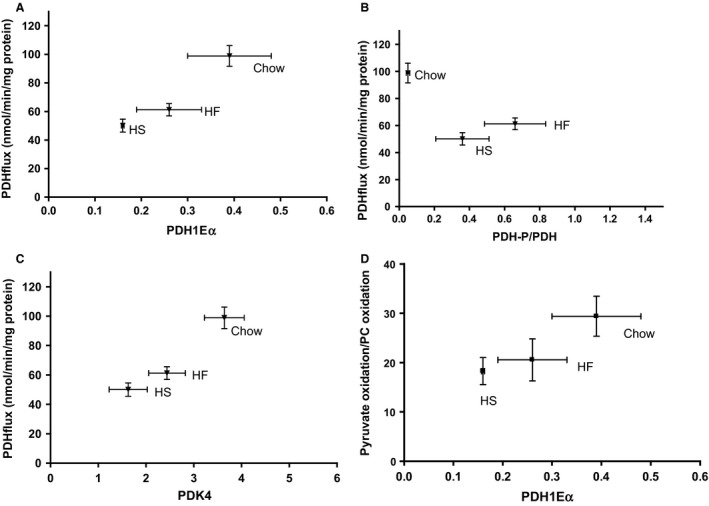
Pyruvate dehydrogenase (PDH) flux versus PDH and PDK4 protein level and PDH phosphorylation in isolated mitochondria from rat skeletal muscle. (A) Total PDH (PDH1E*α*). (B) Degree of PDH phosphorylation (PDH‐P/PDH). (C) Level of Pyruvate dehydrogenase kinase 4 (PDK4). (D) The ratio (PDH oxidation rate(/(*β*‐oxidation rate). All Western blotting data are given in arbitrary units.

**Figure 3:**
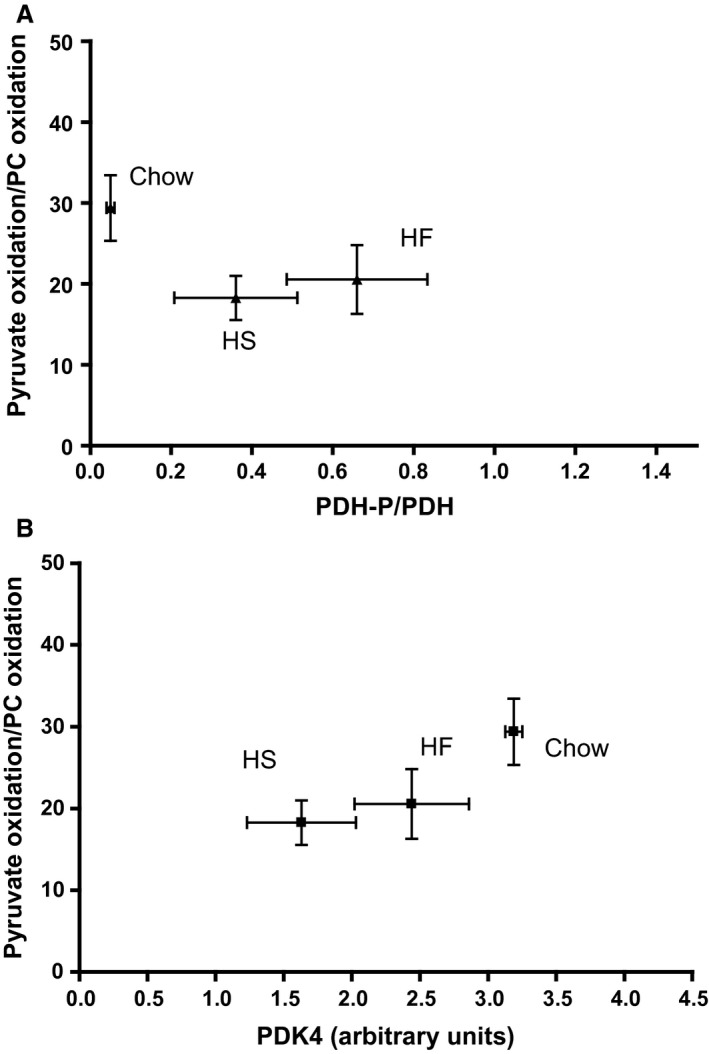
The ratio pyruvate oxidation/*β*‐oxidation rate versus the relative amount of PDH‐P (Fig. 3A) and the PDK4 level (Fig. 3B), as measured in isolated mitochondria from rat quadriceps muscle. Values are means ± SEM,* n* = 5–6.

**Figure 4:**
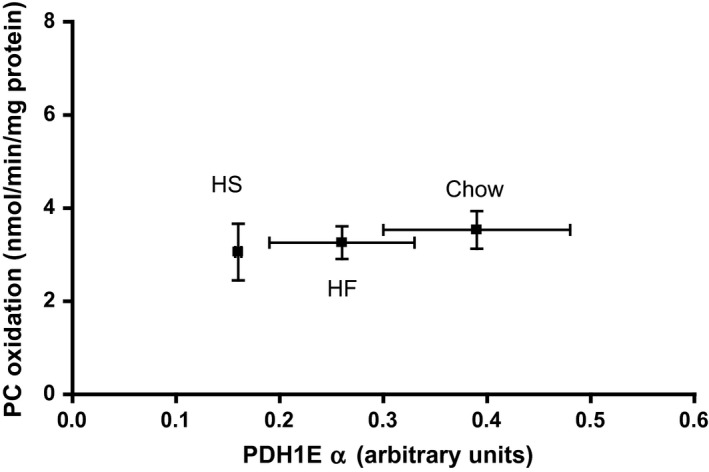
*β*‐oxidation rate versus total PDH level as measured in isolated mitochondria from rat quadriceps muscle. Values are mean ± SEM,* n* = 5–6.

In order to elucidate the mechanism of the observed effects of the HS and HF diets on the PDH flux described above, we measured the amount of total PDH protein, phosphorylated PDH (PDH‐P) as well as the amount of the enzyme known to catalyze this phosphorylation process, the PDH‐kinase‐4 protein (PDK4), by Western blotting as shown in Figures 2A–D and S1. These measurements demonstrate a positive correlation (*P* < 0.05) between the total PDH protein (here displayed by the *PDH1Eα* subunit) and the measured PDH flux (Fig. 2A and Table 2); while the degree of phosphorylation of PDH (i.e., the ratio PDH‐P/(total PDH)) did not correlate with the PDH flux (Fig. 2B). This paradoxical regulatory pattern is also seen in Figure 2C (and Fig. S2, including individual data set) displaying a positive correlation between PDK4 amount and PDH flux. Thus, the decreased PDH flux in isolated skeletal muscle mitochondria brought about by the HF and HS diets does not seem to be mediated via PDH phosphorylation but rather appears to be a function of the total PDH activity. This notion is strengthened by the finding that the flux ratio, pyruvate dehydrogenase/*β*‐oxidation, correlated positively with the total amount of PDH (Fig. 2D), but remained uncorrelated with both PDK4 and PDH‐P (Fig. 3), while the PC oxidation rate was uncorrelated with the amount of total PDH protein (Fig. 4).

## Discussion

In this study we have examined the effects of early life exposure to either a high fat (HF) (60E% lipid) or a high sucrose (HS) (70E% sucrose) diet for 12 weeks after weaning. It may be argued that both diets represent extreme metabolic conditions compared to the balance of substrates normally presented to cell metabolism. It is likely that such metabolic conditions would affect the regulatory mechanisms of the cells. Thus, it is well known that insulin resistance may be induced in liver and skeletal muscle in experimental animal models involving HF and HS diets (Asmann et al. 2006; Befroy et al. 2007; Noland et al. 2009), and many studies indicate that the mitochondrial function are in fact changed when insulin resistance in skeletal muscle is present (Kelley et al. 2002; Asmann et al. 2006; Mogensen et al. 2007; Noland et al. 2009; Jorgensen et al. 2012).

We have therefore investigated if isolated mitochondria from skeletal muscle of rats on a standard rat chow diet displays an interaction between carbohydrate‐ and fat oxidation as seen in vivo (Randle et al. 1963; Hue and Taegtmeyer 2009). And we have investigated if such metabolic interaction is affected by prior exposure of the animal to extreme diets of either high fat or high sucrose.

In other words, we have asked whether isolated skeletal muscle mitochondria change their metabolic flexibility upon long‐term (12 weeks) exposure to a HF and a HS diet.

### Substrate interaction in isolated mitochondria and in vivo (metabolic flexibility)

Muscle mitochondria rely on fatty acids and carbohydrates (in the form of pyruvate) as substrates for energy production. Both substrates supply acetyl CoA to the TCA cycle for further oxidation, and, as first observed by Randle et al. (1963), there is a pronounced interaction between glucose and fatty acid oxidation at the whole body level. This so called *Randle‐effect* [often also loosely referred to as metabolic flexibility (Galgani et al. 2008; Kelley et al. 1999)], is suggested to reflect a dynamic adaptation of the metabolic pathways in response to changed substrate availability. In the present article we observe (Table 2), that the equivalent of a *Randle‐effect* is indeed present in isolated mitochondria from skeletal muscle, that is, a decreased pyruvate oxidation is observed when adding a fatty acid substrate, palmitoyl carnitine (PC) to mitochondria already oxidizing pyruvate. Conversely, we also observe a decreased PC oxidation rate by adding pyruvate to mitochondria already oxidizing PC. Thus, our data demonstrate that metabolic flexibility, similar to in vivo (Randle 1998), exists at the level of the isolated skeletal muscle mitochondria, which defines the phenomenon as a consequence of inherent biochemical network interactions. Thus, we show that the mitochondrial “Randle effect” can be played out without the cytosolic‐mitochondrial interaction and that involvement of malonylCoA does not appear to be necessary.

This metabolic flexibility inherent to muscle mitochondria may be seen as the ability of the PDH and *β*‐oxidation pathways to individually, as well as collectively, to adapt to the TCA flux needed by the actual energy demand of the cell.

The fact that oxygen consumption is roughly doubled when adding pyruvate to skeletal muscle mitochondria oxidizing PC, illustrates another phenomenon, namely that oxygen consumption rate during fatty acid oxidation is set by the *β*‐oxidation capacity and/or the uptake rate of PC into the mitochondria and not by the mitochondrial respiratory capacity. Extending this notion to the whole body level, suggests that maximal energy output from skeletal muscle mitochondria requires the presence of a carbohydrate substrates generating pyruvate as demonstrated in (Rasmussen et al. 2001).

Part of the explanation for the *Randle‐effect* is that the NAD/NADH as well as the CoA/acetylCoA ratio have strong regulatory influence on the PDH flux (Hue and Taegtmeyer 2009). Therefore, it is likely that the PDH flux is key to mitochondrial metabolic flexibility as supported by the observations in Figure 2. Regulation of *β*‐oxidation flux could also be involved in the observed mitochondrial metabolic flexibility, yet the lack of correlation with the PDH flux does not suggest that this is not the case (Fig. 2D). Furthermore, the *β*‐oxidation pathway and succinate dehydrogenase reaction of the TCA cycle may interact since they are sharing the mitochondrial FAD/FADH_2_ redox couple (Jorgensen et al. 2012). For the HS group such interaction is suggested by the fact that with PC as substrate, addition of succinate does not increase mitochondrial oxygen consumption as is normally seen in muscle mitochondria (Jorgensen et al. 2012). Interestingly, such lack of succinate mediated oxygen consumption increase was shown to be lost also in mitochondria from the GK rat model (Type 2 diabetes) (Jorgensen et al. 2012). Other interactions at the level of the CoA/acetylCoA and/or the NAD/NADH ratios may, however, also contribute (Eaton 2002).

### Metabolic flexibility disappears after a high‐fat and a high‐sucrose diet

Our key observation is that metabolic flexibility executed by fat‐carbohydrate oxidation interaction is lost in isolated skeletal muscle mitochondria prepared from animals exposed for 12 weeks in early life to an extreme diet consisting of high fat and even more so to a high‐sucrose diet. What we observe, is the following: For the chow group, the pyruvate oxidation rate is reduced by some 30% when a fatty acid substrate (PC) is added on top of the pyruvate. Conversely, PC oxidation is reduced some 20% by addition of pyruvate. Yet, these effects are modified in mitochondria from individuals with prior exposure to a HS diet and to a lesser extend also a HF diet.

**Figure 5:**
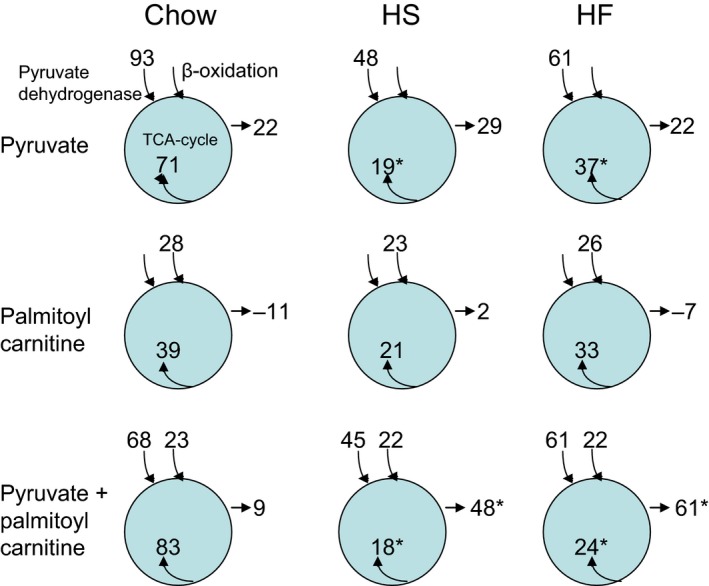
Calculated TCA cycle flux in isolated rat quadriceps mitochondria. Mitochondria were isolated from the quadriceps muscles of the three groups of rats (chow, HS and HF rats) as described in Methods. The three experimental groups are indicated on top of each column in the figure, and the substrate(s) are given to the left of each row. The calculated numbers for TCA input are pyruvate dehydrogenase flux and *β*‐oxidation flux as applicable, and were taken from Table 2. The calculated TCA cycle flux (nmol acetyl‐CoA per mg mitochondrial protein per min) is indicated inside each circle, and represents complete oxidation of acetylCoA to carbon dioxide and water. This number was calculated as: ([total oxygen uptake − oxygen uptake needed for citrate formation]/1.5). Free acetylCoA is assumed to remain constant. Values of total oxygen uptake were taken from Table 1. Substrate oxidation not accounted for by complete oxidation in the TCA cycle is indicated by numbers to the right of each circle, and is given in the same unit as the TCA flux (nmol acetyl‐CoA per mg protein per min). For the sake of clarity, the calculated numbers are given without SD. However, *Indicate significant differences found with respect to the chow group (*P* < 0.05).

In order to get a more clear picture of the metabolic flexibility phenomenon and how it is affected by the HF and HS diets, we have calculated some key flux parameters in the mitochondria, as shown in Figure 5. The calculations are based on the state 3 oxygen consumption and substrate oxidation data (Table 1 and 2, respectively)

With pyruvate as substrate the PDH flux is ~50% reduced in the HS group (93 vs. 48) and ~30% reduced in the HF group (93 vs. 61) compared to control. While the calculated TCA cycle flux with pyruvate as substrate was reduced even more pronounced, ~70% in the HS and ~50% in the HF group. Yet the *β*‐oxidation pathways active with PC as substrate retain an unchanged oxygen consumption and TCA cycle flux. Adding pyruvate and PC together as substrates we observed similar changes as with pyruvate alone as substrate.

Thus, it may be concluded that both the HS‐ and the HF diet for 12 weeks causes a profound decrease in the TCA cycle flux in isolated muscle mitochondria and that the mechanism seems to be a reduced pyruvate dehydrogenase flux with unchanged *β*‐oxidation flux. Accumulation of TCA cycle intermediates are likely when TCA cycle flux is decreased this much, and we have calculated this number as the amount of substrate metabolized that is unaccounted for by the oxygen consumption rate (as explained in the legend of Fig. 5). With pyruvate or PC as the sole substrate, the unaccounted substrate metabolism is unchanged in the HS and HF groups compared with the chow group. While with combined pyruvate and PC as substrates ‐ simulating some form of substrate overload to the mitochondria ‐ there is a significant increase in the calculated unaccounted for substrate of 48 ± 11 and 61 ± 7 nmol C‐2 units per min per mg protein in the HS and HF groups, respectively. This is to be compared with a value in the chow group of only 9 ± 24. (*P* < 0.05).

Qualitative similar observations were reported by Koves et al. (2008) in rats given a high‐fat diet. These authors suggest that the result was linked to skeletal muscle insulin resistance. In the present experiment, however, we see glucose intolerance in the HF group but not in the HS group (Fig. 1), while none of the groups show insulin resistance. Nevertheless there were significant TCA cycle flux decreases, that is, ~80% and ~50% in the HS and HF groups, respectively (Fig. 5). This remains unexplained but may be related to the fact that the fructose present in the HS diet does not stimulate insulin secretion from the beta‐cells (Curry 1989).

Taken together, our observations suggest that the interaction of fatty acid‐ and carbohydrate oxidation in normal isolated skeletal muscle mitochondria is to a large extend executed by the adaptation of the PDH flux to the fat/carbohydrate substrate availability to the muscle mitochondria. The mechanism is a pronounced diet induced decrease of the total, rather than the dephosphorylated, form of the PDH protein as demonstrated in Figure 2A by a twofold decrease of the total PDH activity after a 12 week HS diet, and a somewhat smaller loss of activity after the HF diet. Similar changes were observed for citrate synthase activity, suggesting that the activity of PDH and TCA cycle enzymes per mg of total mitochondrial protein is lower in mitochondria from HS and HF fed compared with chow fed rats. Whereas this does not seem to be the case for enzymes of the *β*‐oxidation pathway, as judged by the flux data reported in Figure 4 and Table 2.

Overall our findings indicate that the interaction between the *β*‐oxidation‐ and PDH‐flux normally seen in skeletal muscle mitochondria is operating via an adaptation of the PDH flux with little or no change of the *β*‐oxidation flux. However, this regulatory property is lost after exposure to the extreme diets and is mechanistically caused by a major reduction in the level of total PDH (and a similar reduction in PDH flux) combined with an even larger decrease in the TCA cycle flux, as demonstrated in Figure 5. These effects are more pronounced with the high‐sucrose than with the high‐fat diet.

Thus, the obesogenic diets as applied here (which are in fact not unrealistic compared to diets eaten on a regular basis by large groups of human beings), have serious effects on the metabolic performance of skeletal muscle mitochondria, while this is not the case in brain mitochondria after a high fat diet (Jørgensen et al. 2015). Importantly however, at this stage it is not known whether the observed effects are in fact reversible upon a change of diet from high fat or high sucrose back to normal chow diet.

## Conflict of Interest

None declared.

## Data Accessibility
